# Determining the porous structure for optimal soft-tissue ingrowth: An *in vivo* histological study

**DOI:** 10.1371/journal.pone.0206228

**Published:** 2018-10-29

**Authors:** Mukai Chimutengwende-Gordon, Robert Dowling, Catherine Pendegrass, Gordon Blunn

**Affiliations:** John Scales Centre for Biomedical Engineering, Institute of Orthopaedics & Musculoskeletal Science, University College London, Royal National Orthopaedic Hospital Trust, Brockley Hill, Stanmore, Middlesex, United Kingdom; Universite de Technologie de Compiegne, FRANCE

## Abstract

The success of osseointegrated transcutaneous prostheses depends on a soft-tissue seal forming at the skin-implant interface in order to prevent infection. Current designs include a flange with drilled holes or a subdermal barrier with a porous coating in an attempt to promote soft-tissue attachment. However, the soft-tissue seal is not reliably achieved despite these designs and infection remains a significant problem. This study investigated soft-tissue integration into fully porous titanium alloy structures with interconnected pores. The study aimed to determine the effect of altering pore and strut size combinations on soft-tissue ingrowth into porous titanium alloy structures *in vivo*. It was hypothesized that implants with a more open porous structure with larger pore sizes would increase soft-tissue ingrowth more than less open porous structures. Porous titanium alloy cylinders were inserted into sheep paparaspinal muscles (n = 6) and left in situ for four weeks. A histological assessment of soft-tissue ingrowth was performed. Percentage soft-tissue pore fill, cell nuclei density and blood vessel density were quantified. The results showed that larger pore sizes were supportive of soft-tissue ingrowth. A structure with a pore size of 700μm and a strut size of 300μm supported revascularisation to the greatest degree. A flange with this structure may be used in future studies of osseointegrated transcutaneous prostheses in order to enhance the soft-tissue seal.

## Introduction

In the United Kingdom, the number of new referrals to prosthetic services annually has increased from 4957 in 2007 to 5988 in 2011[1]. Conventionally, amputees are fitted with a socket, to which an artificial limb is attached. However, the stump-socket interface may cause problems that restrict socket use. Uneven pressure distribution over the stump may lead to pain, tissue abrasion, pressure sores, necrosis and limb disuse. Additionally, the unnatural microbial environment allows the development of infection [2,3]. Skin-penetrating osseointegrated implants are an alternative way of attaching the external prosthetic device to the body [4]. They aim to provide a stable attachment for prosthetic limbs, allowing the transfer of weight through the skeleton, eliminating the pressure on the stump-socket interface [5]. Crucially, functionality of these devices requires a tight soft-tissue seal at the transcutaneous interface, by promoting attachment of the dermal and epithelial tissues [5,6,7]. Osseointegration has been successfully achieved, however infection due to failure of the soft tissues sealing the transcutaneous interface hamper clinical longevity [8].

Titanium alloy (Ti6Al4V) is frequently used in orthopaedics because it is highly biocompatible and corrosion resistant [9]. Implants, in combination with hydroxyapatite (HA) coatings, are successful as endoprostheses requiring bony fixation, however these implants do not promote soft-tissue attachment or ingrowth [10]. Porous structures can act as a mechanical scaffold to aid cell attachment for implant biomaterials. Inter-pore connections allow cells to in-grow through implants. This benefits load-bearing implants, promoting osseointegration through bio-fixation and mechanical attachment of bone. High volumetric porosity of implants can also positively influence biological tissue integration at the soft-tissue level. The permeability of an open structure can facilitate transport of body fluid through the implant promoting ingrowth of well-vascularised soft tissue in a short period of time [9, 11, 12, 13]. Osseointegrated transcutaneous implants have been modelled on the deer antler pedicle. These naturally occurring structures support a soft-hard tissue interface due the porous nature of the subcutaneous bone allowing the soft tissues of the dermis and epidermis to infiltrate and attach forming a tight seal. This prevents any motion between the soft tissue and the bone that may occur if these tissues were not attached to the bone surface. Measurement of the pores size in deer antlers suggests a mean pore diameter of 217μm (+/-19.07μm) [6]. Current models of osseointegrated transcutaneous prostheses have used a flange with drilled holes or a porous-coated subdermal barrier rather than a fully porous design with interconnected pores [5, 14].

Research on integration of a variety of soft tissues types has been conducted with conflicting evidence relating to optimal pore sizes [5, 15, 16, 17, 18]. This study assessed soft-tissue integration into a range of porous Ti6Al4V implants fabricated by electron beam manufacturing, with pore sizes up to 1000 μm in diameter, inserted into the paraspinal muscles in an ovine model. The aim of the study was to determine the optimum pore and strut size combination to promote soft-tissue integration. It was hypothesized that more open porous structures would lead to greater soft-tissue ingrowth and vascularisation. The optimum porous structure identified may be applied to the subdermal flange of osseointegrated transcutaneous prostheses to support dermal tissue integration in order to seal the interface and reduce the risk of infection.

## Materials and methods

Cylindrical, surgical grade Ti6V4Al implants, 20mm in height and 10mm in diameter, were manufactured using Electron Beam Manufacturing (EBM) (EOS GmbH Electro Optical Systems, Germany). Nine implant groups were tested using different combinations of pore and strut sizes (Table 1). These implants were surgically implanted into the paraspinal muscles of skeletally mature female sheep.

**Table 1:** Implant group dimensions.

Group	Pore Size/ μm^2^	Strut Size/ μm^2^
1	1000	400
2	1000	200
3	700	400
4	700	300
5	700	200
6	500	400
7	500	300
8	500	200
9	200	300

Table showing pore and strut size combinations for each implant group.

## Ethical statement

The project and animal facilities were approved by the United Kingdom Home Office Licensing Authority. The study was conducted in accordance with the United Kingdom Animal Scientific Procedures Act 1986 and the procedures were performed under the Home Office Project Licence (70/6964).

## Animal husbandry

One week prior to surgery six adult female sheep (cross bred mules), three to four years of age, were group housed on straw in a large pen located undercover with up to 12 animals in each group. The animals were sourced from the Royal Veterinary College, Hertfordshire, United Kingdom. One day prior to surgery animals were transferred to an individual pen in the same barn. Analgesia was maintained with fentanyl transdermal patches (75μm/hour) (Duragesic, Janssen Pharmaceuticals, NJ, USA) applied to the shaved operative site 12 hours preoperatively. Animals were starved for 12 hours prior to surgery. Postoperatively, the animals were returned to the pen, kept on straw and given food and water *ad libitum*. The fentanyl patch was changed three days after applying the first patch, providing analgesic cover for five days post-operatively. Animals were assessed twice daily by an experienced stock-man and were reviewed by a veterinary surgeon twice a week. Criteria to assess animal health and well-being were vital observations i.e. heart rate, respiratory rate and temperature as well as monitoring for signs of animal behaviour that could indicate pain or distress e.g. lethargy, inappetence and a change in facial expression, stance or lying position. After a minimum of four days recovery, animals were returned to the group.

### Surgical procedure

#### Premedication and anaesthesia

0.2 mg/kg of 2% xylazine (Bayer HealthCare, Berkshire, UK) was administered as premedication to each animal. Anaesthesia was induced with 2mg/kg of intravenous Ketamine Hydrochloride (Ketaset, Fort Dodge Animal Health Ltd., UK) and 2.5mg of Midazolam (Hypnovel, Roche Products Ltd., UK) and maintained with 2% inhaled isofluorane. 5ml of Cefalexin Ceporex (Schering-Plough Animal Health, UK) was administered to achieve antibiotic prophylaxis.

#### Operative details

The anesthetised animals were positioned lying prone. The spinal region was shaved pre-operatively approximately from the level of the twelfth thoracic vertebra to the sacrum. The width of the shaved area was approximately 15 cm positioned centrally over the spine. The shaved area was prepared with povidone iodine solution and then with antiseptic chlorhexidine solution. 2 cm longitudinal incisions were made through the skin, subcutaneous fat and fascia paraspinally and the cylinders were implanted within the paraspinal muscles. 3–0 vicryl sutures were used to close the fascia and subcutaneous tissues and a continuous subcuticular 3–0 vicryl suture was used to close the skin. Four implants were implanted through separate incisions on both sides of the spine.

#### Removal of implants

Four weeks post implantation; animal subjects were euthanized by intravenous injection of 0.7mg/kg Sodium Pentobarbitone (Pharmasol Ltd., Andover UK). All implants were dissected out en bloc and placed in 10% formal saline for one week.

### Histological procedure

The samples were serially dehydrated with industrial methylated spirits and defatted in chloroform over a five-week period. Hard Grade Acrylic Resin (London Resin Company Ltd., London, UK) was given time to penetrate each sample before being embedded. Transverse sections were made through the centre of each implant using an Exact E310 diamond edged band saw (Mederex, Frome, UK). Sections were ground evenly using Exact-Micro-Grinding System (Mederex, Frome, UK) to a thickness of 100μm. All sections were stained with Toluidine Blue, photographed with Carl Zeiss photomicroscope (KS300, Carl Zeiss, Oberkochen, Germany) and digital image processing software (Axiovision Rel 4.5, Carl Zeiss, Oberkochen, Germany).

### Histological analysis

The cross-section was then divided in three zones: both outer edges (zones 1 and 2) and the central region (zone 3). This was to ensure data was collected across the entire implant. Each outer zone had data collected from two separate points and the average calculated. One data collection point was taken in the central zone. A semi-quantitative percentage score (%Fill) was assigned at each data point, based on soft tissue outside the implant. A percentage for soft-tissue fill was assigned based on the percentage of the pore that was filled with soft tissue as well as taking into account the density, quality and maturity of the tissue within the pore compared with the appearance of the tissue outside the implant. A score of 100% would be assigned to a pore infiltrated completely with dense, well-ordered fibrous-connective tissue that was in intimate contact with the pore edge. 0% would indicate a pore devoid of soft tissue. This was scored by two independent assessors. These data were substantiated with a count of blood vessels (BV/mm^2^) to assess re-vascularisation and a cell nuclei count (CN/mm^2^) to assess the re-cellularisation.

### Statistical analysis

The data were analysed using SPSS, version 21.0 for Windows (Chicago, US). Nonparametric tests were performed. The Kruskall Wallis test was used to determine if differences existed between the groups. Pair-wise Mann-Whitney tests were used to determine differences between two porous structures. A p value of less than 0.05 was considered to be a statistically significant result. The intraclass correlation coefficient for agreement between the two assessors of the percentage soft-tissue semi-quantitative score was calculated.

## Results

All the quantitative data collected is summarised in Table 2. Of particular interest are the data collected from zone 3, the innermost zone. The data collected from this zone gives the strongest indication of soft-tissue ingrowth throughout the entire implant.

**Table 2:** Median values for all collection variables for each group.

Group	Pore Size/ μm^2^	Strut Size/ μm^2^	%fill Zone1	%fill Zone2	%fill Zone3	CN/mm^2^ Zone1	CN/mm^2^ Zone2	CN/mm^2^ Zone3	BV/mm^2^ Zone1	BV/mm^2^ Zone2	BV/mm^2^ Zone3
1	1000	400	100	98.50	100	3440.50	2846	3586.50	4	5.20	2
2	1000	200	100	100	100	5059	4527	5000	8	10	8
3	700	400	40	35	25	1190	700	584.50	4.50	1	2.5
4	700	300	100	95	100	1389	1243	1306	15	6	11
5	700	200	88	87.50	90	815	604	551.50	3	1.5	0
6	500	400	27.50	22.50	10	250	268.50	251	0	0	0
7	500	300	60	45	40	1390	1199	821	9	4	0
8	500	200	60	63	60	890	645	720	8	4	4
9	200	300	N/A	N/A	N/A	N/A	N/A	N/A	N/A	N/A	N/A

Median values for percentage soft-tissue fill, cell nuclei density and blood vessel density for each implant group.

### Percentage soft-tissue fill

**Fig 1:**
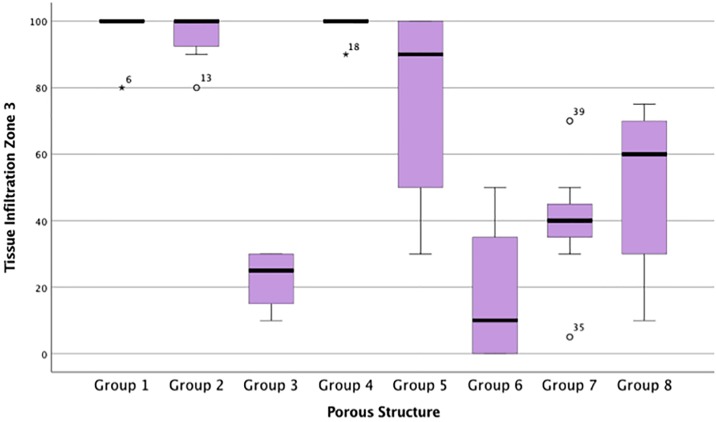
Percentage soft-tissue fill for each porous implant group within the innermost zone, Zone 3. Box plot demonstrating the percentage soft-tissue fill for each porous implant group within the innermost zone, Zone 3.

**Fig 2:**
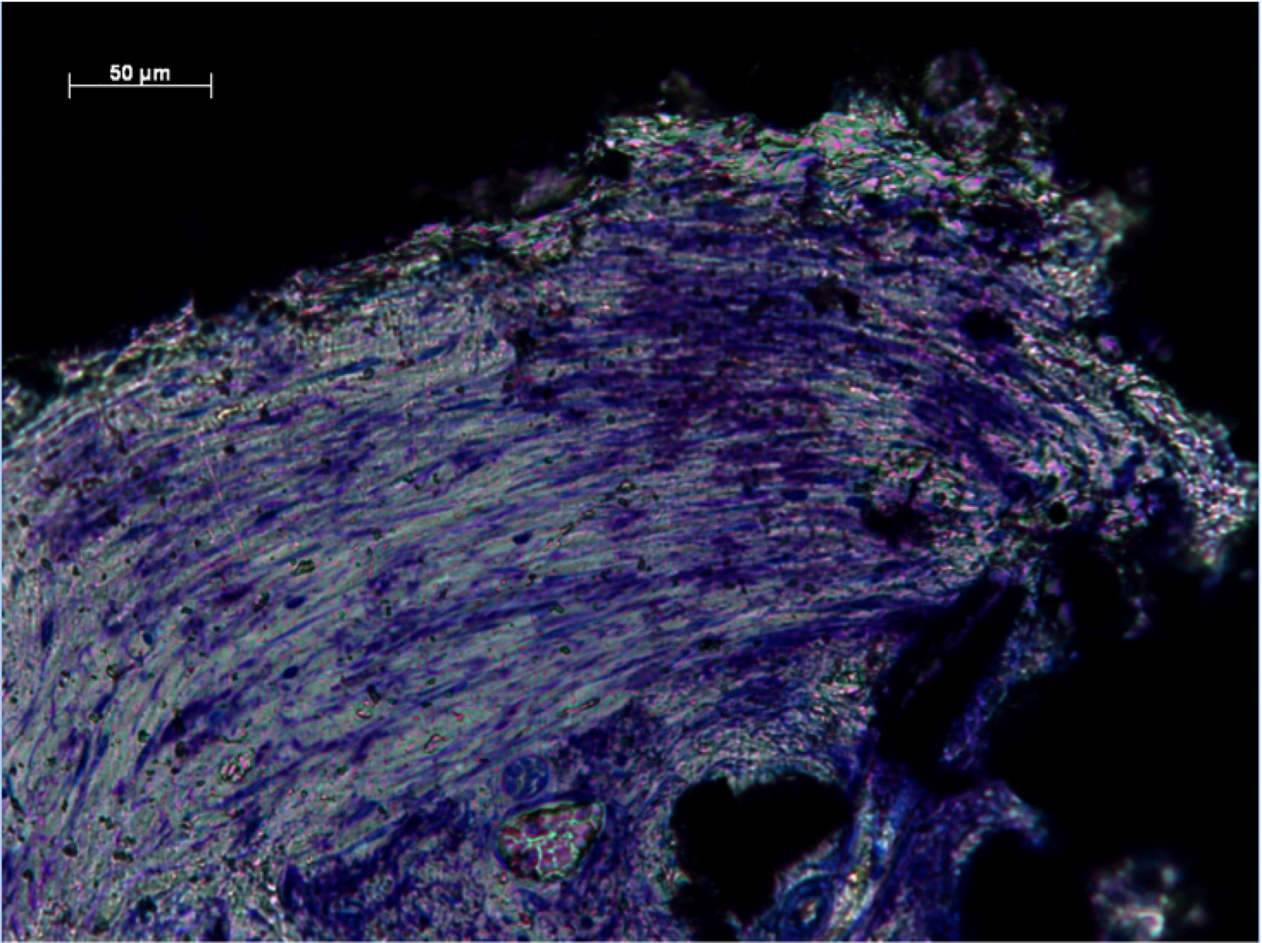
Soft-tissue infiltration in Zone 1 of Group 4. Histological section of Group 4 (Zone 1) demonstrating dense, well-ordered soft-tissue infiltration.

**Fig 3:**
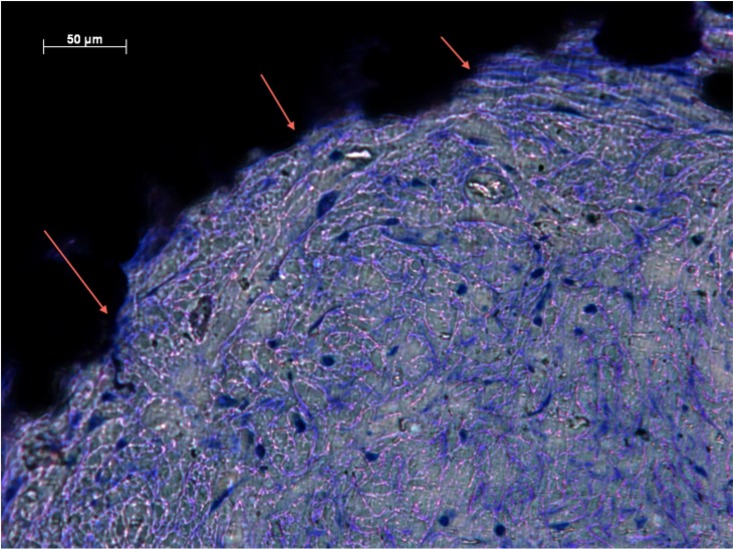
Soft-tissue infiltration in Zone 2 of Group 1. Histological section of Group 1 (Zone 2) demonstrating intimate contact (arrows) between pore edge and soft tissues.

**Fig 4:**
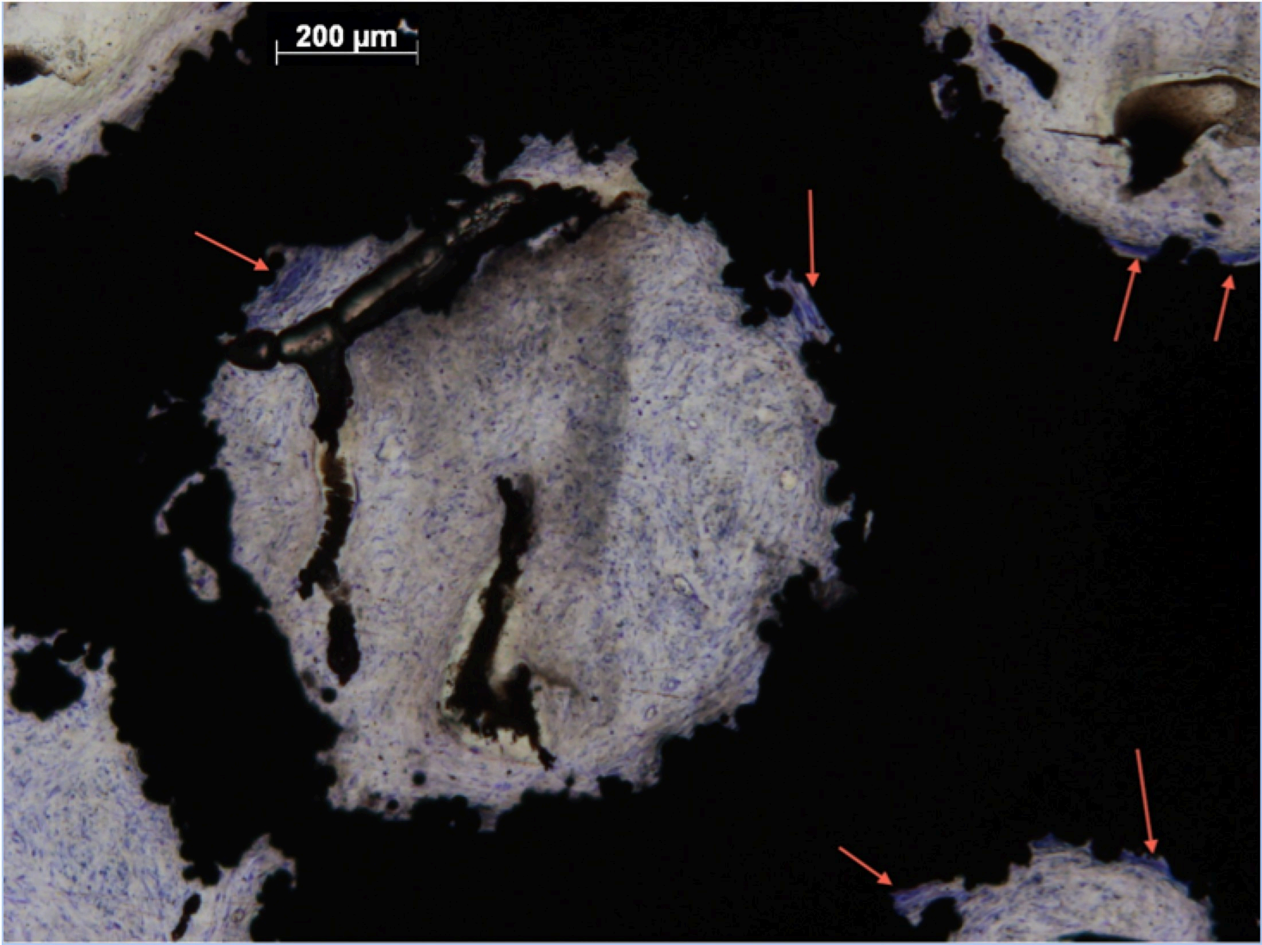
Soft-tissue infiltration in Zone 3 of Group 2. Histological section of Group 2 (Zone 3) demonstrating soft tissue preferentially depositing collagen around interconnecting struts (arrows).

**Fig 5:**
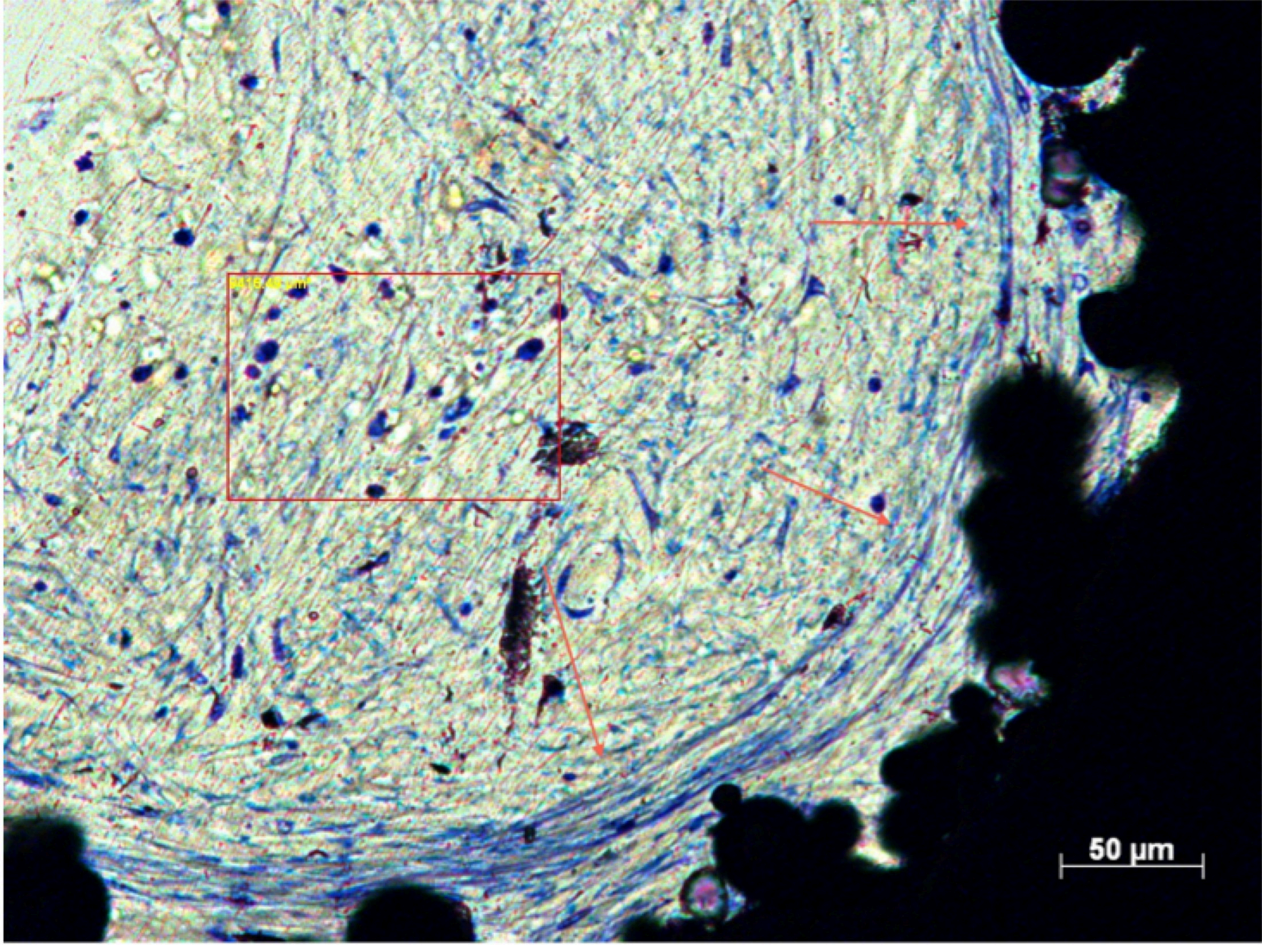
Soft-tissue infiltration in Zone 1 of Group 3. Histological section of Group 3 (Zone 1) demonstrating preferential deposition of collagen around interconnecting struts (arrows) with distinct cell nuclei populating the implant pore.

%Fill data from all zones were not from the same population distribution (p <0.05). The intraclass correlation coefficient for agreement between the two independent assessors for the percentage soft-tissue fill semi-quantitative score was 0.870 (95% confidence interval 0.822 to 0.906). Groups 1, 2 and 4 observed highest %Fill (Fig 1 and Table 2). There were significant differences between groups 1 and 3 (p<0.05), and 3 and 4 (p<0.05). No significant differences were observed between groups 1 and 2 (p = 0.176), or 4 and 5 (p = 0.205). Dense, well-ordered soft tissues filled the entirety of the pores of implants from Groups 1, 2 and 4. Extensive ingrowth across the entire implant was observed with little open space visible. Intimate contact at the tissue implant interface suggested stable integration (Figs 2 and 3). Moreover, preferential collagen deposition appeared evident around interconnecting struts in these implants groups (Figs 4 and 5). Soft-tissue ingrowth was not observed with implants from Group 9.

### Cell nuclei density

**Fig 6:**
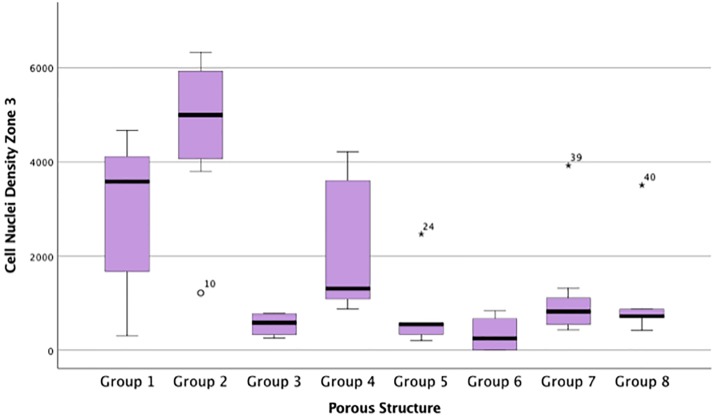
Cell nuclei density for each porous implant group within the innermost zone, Zone 3. Box plot demonstrating the cell nuclei/mm^2^ for each porous implant group within the innermost zone, Zone 3.

**Fig 7:**
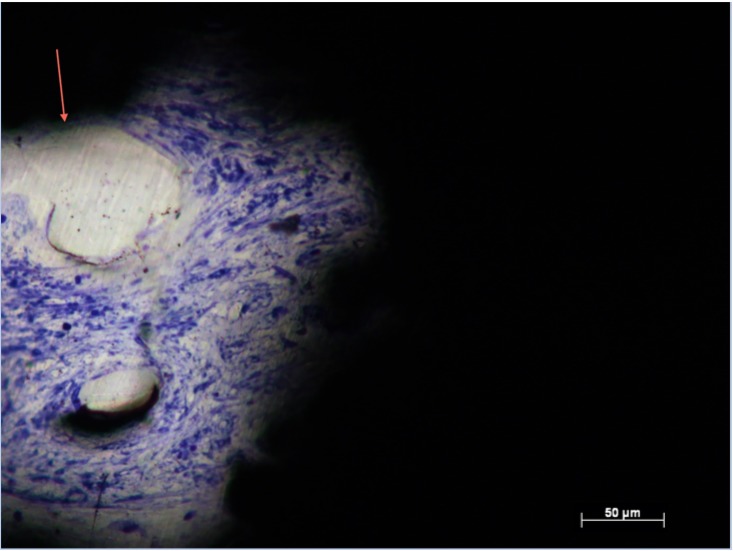
Cell density in Zone 1, Group 8. Histological section of Group 8 (Zone 1) demonstrating less dense soft tissues with poor contact (arrows) between pore edge and soft tissues.

**Fig 8:**
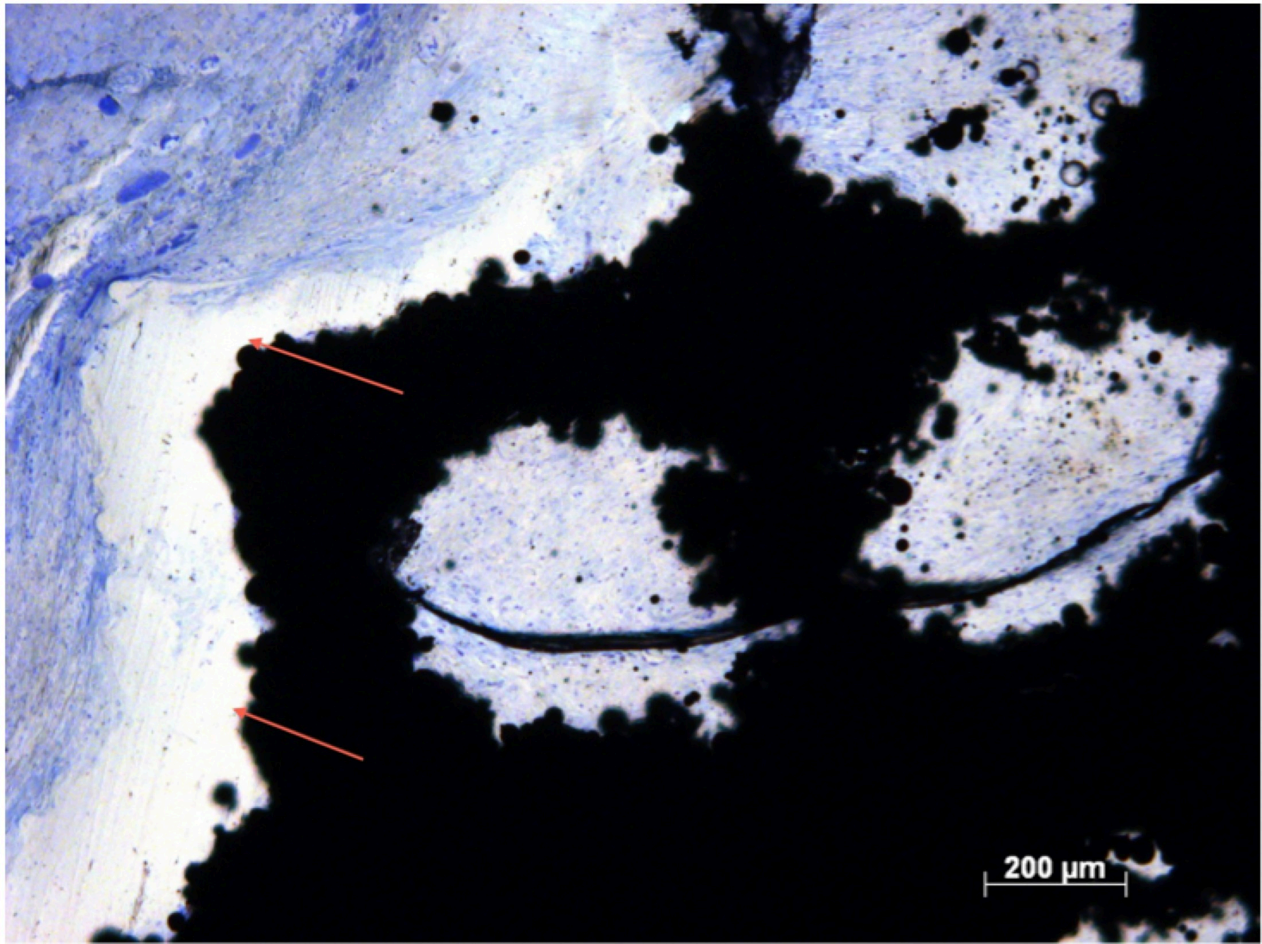
Cell density and blood vessel formation in Zone 1, Group 7. Histological section of Group 7 (Zone 1) demonstrating less dense soft tissue with poor contact (arrows) between pore edge and soft tissues.

**Fig 9:**
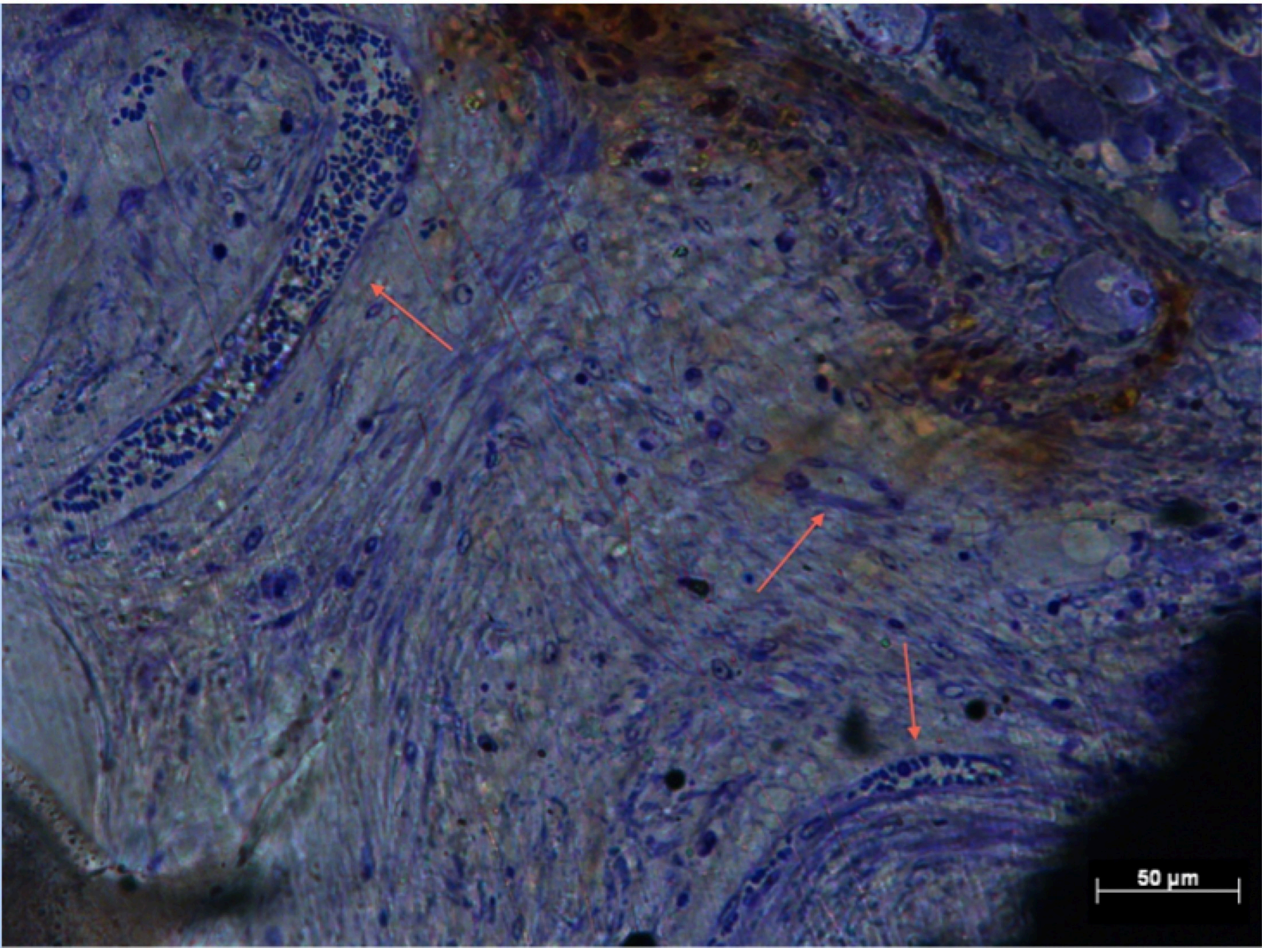
Cell density in Zone 1, Group 4. Histological section of Group 4 (Zone 1) demonstrating dense, well ordered soft tissue with extensive blood vessel formation (arrows).

CN/mm^2^ data from all zones were not from the same population distribution (p <0.05). CN/mm^2^ data from the inner most zone, zone 3 suggested similar relationships as %Fill data (Table 2). Groups 1, 2 and 4 facilitated the highest CN/mm^2^. Group 2 implants demonstrated significantly greater CN/mm^2^ compared with all other Groups (p < 0.05) (Fig 6). Large numbers of individual cells populated pores across the entire implant in Groups 1, 2 and 4, in support of the quantitative data (Table 2, Figs 5 and 6). Figs 7 and 8 show less dense soft tissue. These observations were associated with implant groups with a less open structure, which also corresponded to those with lower CN/mm^2^ (Table 2). Tissue integration within these groups showed gaps with little interface formation between tissue and pore edge (Fig 7). Fig 9 shows dense soft tissue within the pore of an implant from Group 4.

### Blood vessel density

**Fig 10:**
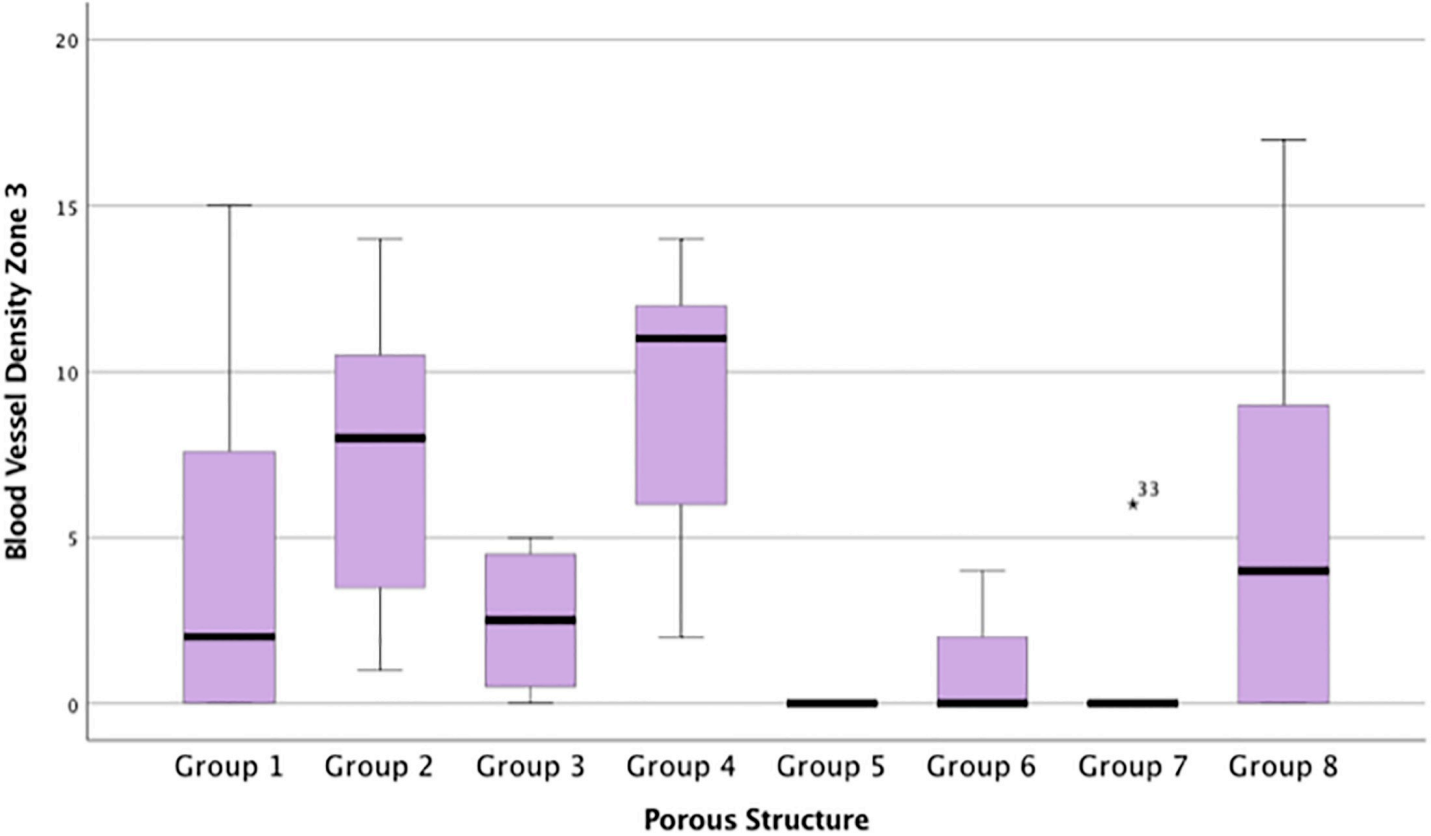
Blood vessel density for each porous implant group within the innermost zone, Zone 3. Box plot demonstrating the blood vessels/mm^2^ for each porous implant group within the innermost zone, Zone 3.

The data for BV/mm^2^ across all zones were not from the same population distribution (p <0.05). Group 2 and 4 demonstrated greater re-vascularisation in zone 3 compared with all other implants (Fig 3). The number of BV/mm^2^ in Group 4 was significantly higher compared with Group 5 and 7 (p <0.05). Significantly higher numbers of BV/mm^2^ were observed in Group 2 compared with Group 5 (p<0.05). Group 4 was associated with significantly higher BV/mm^2^ compared to all other groups in zone 1 (Fig 10). Blood vessels were visible with endothelial nuclei punctuating in a ring creating a lumen. More of these vessels were observed in more open structures associated with Groups 2 and 4 (Table 2, Fig 10), suggestive of a capillary network throughout the implant. No necrotic tissue or degradation was evidenced (Fig 8). Re-vascularisation was not observed throughout implants in Groups 5, 6, and 7, supportive of the BV/mm^2^ data (Table 2, Fig 9).

## Discussion

Subcutaneous soft tissue infiltrated throughout all porous implants groups after four weeks *in vivo* except Group 9. It has previously been hypothesised that the anatomical and physiological characteristics associated with soft tissues would require a more open structure to maintain viable tissue compared with bone tissues [15]. Group 9 pore diameter was 250μm and therefore may have been too small to allow tissue infiltration. This is in keeping with the findings of LaBerge et al [16] who observed fibrous encapsulation of implants with pore diameters of 300μm rather than infiltration. However, in contrast to our results, others have observed soft tissues infiltrating structures with similarly small diameters [15, 17, 19]. Bone tissue has also been shown to infiltrate into pores below this diameter [20, 21]. Ponader et al [9] suggest successful infiltration with bone tissue can be achieved with a pore diameter of 450μm, which is significantly smaller than the 1000μm and 700μm diameter pores successful in this study. By comparison, there are obvious differences in tissue type between this study and ours. The authors did discuss that there could be a minimum size requirement to facilitate the influx of tissue. A much larger pore diameter may support vascularisation, allowing tissue perfusion with oxygen and promoting osteogenesis [9]. This is important particular for extensive porous structures where tissue migration into the centre of the implant is required. The factors governing bone ingrowth into porous coatings on the surface of orthopaedic implants where minimal ingrowth is required, may be very different from those associated with tissue ingrowth deep into extensively porous structures and may be different with different tissue types. A contributing factor that may explain these conflicting results is the relative movement of the implant structures *in vivo*. Bobyn et al secured implants into the subcutaneous soft tissue by suturing, thereby decreasing relative movement in situ and allowing cell and tissue infiltration into significantly smaller pore dimensions [17]. This was not performed in our study, nor by LaBerge et al [16] where little or no soft-tissue infiltration was observed with smaller dimensions. It is possible that when implants are incorporated into an osseointegrated prostheses, the degree of soft-tissue tissue infiltration may be increased, as the bone-anchored stem will limit relative implant movement *in vivo*.

Pore diameters greater than 700μm supported extensive tissue infiltration compared with less open structures. Groups 1, 2 and 4 (pore size:strut size being 1000:400, 1000:200 and 700:300 respectively) demonstrated superior tissue infiltration across their entire structure when compared with other groups The open structure in these implant groups facilitated the invasion of blood vessels into the structure which supported early attachment of cells. Group 3 implant structures appeared not to follow the trend, observing lower soft-tissue infiltration despite possessing a large pore diameter of 700μm. The interconnecting strut dimension of 400μm in Group 3 implants may have detrimentally influenced tissue infiltration due to the fact that the larger strut size reduces the volumetric porosity preventing migration between pores and limiting the total volume of available pore space for the entire implant.

Hacking et al observed soft-tissue infiltration throughout porous tantalum implants at four weeks [15]. They also noted an increase in tissue infiltration over time and an associated increase in attachment strength by mechanical testing. If the degree of tissue infiltration is directly linked to tissue attachment strength, it suggests that the dimensions of Groups 1, 2 and 4 in our study would support increased soft tissue attachment strength. A patient with an osseointegrated implant reports near normal functionality as a result of the treatment [7]. This increases the physical demand on the soft tissue implant interface and subsequently, a device that supports increased tissue attachment strength would be more favourable.

Collagen appeared to preferentially deposit at the pore edges and interconnecting struts, extensively in Groups 1, 2 and 4. These features are indicative of soft-tissue integration. Albrektsson et al define tissue implant integration as the interaction of collagen and metal oxide [22]. Howe et al also noted these features in collagen deposition around pore edges at the bone implant interface [20]. Using a biomimetic example of a bone percutaneous interface, Pendegrass et al showed collagenous ‘Sharpey-like’ fibres anchoring dermal tissue to the deer antler pedicle where the soft-tissue interface is linked with the bone by collagen fibres that pass from the bone into the soft tissue [6].

The cellular component of the many soft tissues, especially the subcutaneous tissue in this study, are fibroblasts [23] and are identifiable in the histological analysis as the Toluidine blue stains nucleic acids within the cell nuclei. Groups 1 and 2 showed significantly increased cell nuclei density compared with other implant groups. Group 4 also exhibited a high level of cell nuclei density compared with Group 3 implants, significantly so in zone 3, despite both possessing the same pore dimensions. The increases in cell nuclei density may result in an increase in collagen deposition, as discussed earlier, because if the majority of cells are fibroblasts they would be expected to produce an extracellular matrix rich in collagen [23]. The observed increases in cell nuclei density (seen in Groups 1 and 2, and to a lesser degree 4) can be linked to the structural dimensions possessed by the implant groups. The structural environment may support the movement of fibroblasts across the entire implant. Fibroblast migration across porous titanium alloy has been demonstrated, in limited fashion, *in vitro* [24] and can be controlled *in vivo* by decreasing the free space between pores [25]. Cell nuclei densities between Zone 1 and 3 were not significantly different for Groups 1, 2 or 4. This suggests a more uniform movement of cells across the entire implant structures.

Blood vessel formation within the pores was observed across the entire implant structure in Groups 1, 2, 3, 4 and 8. Groups 2 and 4 saw the highest blood vessels densities, with no significant differences between zones. The 400μm strut dimension, of Groups 1 and 3 was associated with reduced vascularisation in these implant groups by lowering the overall volume within the implant, taking up free space. Re-vascularisation provides nutrients and facilitates the removal of metabolic products, supporting healthy soft tissue [19]. Porous titanium implants with much smaller inter-porous spaces (150μm) fail to support blood vessel formation [18]. Long-term *in vivo* success relies on the early formation of an extensive vascular network [26]. Hypoxic conditions associated with the lack of blood vessels may have detrimentally affected soft-tissue ingrowth within less open structures, conversely, the more open structures of implant Groups 2 and 4 allow soft tissue to flourish as a well-oxygenated environment was present.

The novel aspect of this study is that it quantifies soft tissue infiltration into a series of extensively porous structures made by EBM. The EBM technique employed in this study created continuous 3-D porous structures rather than a porous coating consisting of a single layer [16] or several [17] layers of pores. This study has assessed the effect of altering pore and strut size and has quantified the tissue within the implant structures, whereas other studies have generally qualitatively described this. It could be argued that the use of a semiquantitative assessment of percentage soft-tissue fill is a limitation of this study as different assessors could potentially interpret findings differently. In order to estimate the likelihood of this, the assessment was carried out by two independent observers. The intraclass correlation coefficient of 0.870 for agreement between the two assessors indicated that the semi-quantitative score had a high level of interobserver reliability. Furthermore, quantitative assessments of cell nuclei density and blood vessel density were carried out. A further limitation of this study is that mechanical testing on the strength of tissue attachment to the implant was not performed, whereas other groups have assessed this [15,16,17]. Mechanical strength of attachment positively correlates with pore size [17] and time *in vivo* [16]. Therefore it is postulated that in the groups where significant increases in subcutaneous soft-tissue infiltration and vascularisation have been observed, it is likely that attachment strength would also be improved.

## Conclusions

The results from this study allow soft-tissue infiltration to be optimised as a function of pore and strut diameter. These implant dimensions were made possible by the EBM process, which would otherwise be unachievable in our laboratory. Group 4 exhibited significantly higher blood vessel densities across the entire implant structure compared with other implant groups. The formation of blood vessels, re-vascularising the soft tissues surrounding the skin implant interface would be expected to be beneficial in the long-term stability of the skin seal. Thus, the incorporation of a porous flange structure, with 700μm pore and 300μm strut dimensions to an ITAP device may provide an opportunity to control the soft tissue interface and in turn develop a stable, biological seal. The ultimate goal for osseointegrated transcutaneous devices is clinical longevity and the seal is of paramount importance to prevent infection of the soft tissues adjacent to the implant. Infection at the interface between the skin and the implant has the potential to track down to bone where it may cause osteomyelitis and necessitate the removal of the implants with disastrous consequences for the patient. The use of porous structures to encourage soft-tissue ingrowth requires long-term *in vivo* assessment and ultimately evaluation in clinical trials to characterise the transcutaneous implant interface.

## Supporting information

S1 Table Data for soft-tissue integration all zones: Median Values and (95% confidence intervals). (DOCX)

S2 Table Zone 1—Percentage Fill p values. (DOCX)

S3 Table Zone 2—Percentage Fill p values. (DOCX)

S4 Table Zone 3—Percentage Fill p values. (DOCX)

S5 Table Zone 1—Cell nuclei count/mm^2^ p values. (DOCX)

S6 Table Zone 2—Cell nuclei count/mm^2^ p values. (DOCX)

S7 Table Zone 3—Cell nuclei count/mm^2^ p values. (DOCX)

S8 Table Zone 1—Blood vessel count/mm^2^ p values. (DOCX)

S9 Table Zone 2—Blood vessel count/mm^2^ p values. (DOCX)

S10 Table Zone 3—Blood vessel count/mm^2^ p values. (DOCX)

S11 Table Correlations. (DOCX)

S12 Table Intraclass correlation coefficient data for semi-quantitative percentage soft-tissue fill score. (DOCX)
