# Supplementary material for: Determining the porous structure for optimal soft-tissue ingrowth: An in vivo histological study
Source: PLoS One. 2018 Oct 29;13(10):e0206228. doi: 10.1371/journal.pone.0206228 (PMC6205611; doi:10.1371/journal.pone.0206228)
Supplement: S1 Table — (DOCX) [file pone.0206228.s001.docx]

**S1 Table. Data for soft tissue integration all zones: Median Values and (95% confidence intervals)**

| **Implant** | **Percentage soft tissue fill** | | | **Cell nuclei/mm^2^** | | | **Blood vessel/mm^2^** | | |
| --- | --- | --- | --- | --- | --- | --- | --- | --- | --- |
|  | Zone 1 | Zone 2 | Zone 3 | Zone 1 | Zone 2 | Zone 3 | Zone 1 | Zone 2 | Zone 3 |
| P1000 S400 | 100  (constant) | 98.5  (95.23 to 100.44) | 100  (84.89 to 107.11) | 3440.5  (1407.93 to 4453.40) | 2846  (800.64 to 4397.02) | 3586.5  (1246.15 to 4728.85) | 4  (1.52 to 9.44) | 5.20  (0.73 to 13.4) | 2  (-1.77 to 10.64) |
| P1000 S200 | 100  (97.54 to 101.03) | 100  (94.22 to 101.50) | 100  (87.94 to 102.06) | 5059 (3330.45 to 6312.70) | 4527 (2402.37 to 5439.06) | 5000 (3004.61 to 6293.96) | 8  (4.28 to 10.29) | 10  (4.52 to 12.05) | 8  (2.83 to 11.74) |
| P700 S400 | 40  (11.84 to  78.66) | 35  (18.61 to 53.89) | 25  (7.27 to 37.73) | 1190  (-409.57 to 2953.57) | 700  (-392.4 to 2157.9) | 584.5 (131.82 to 970.68) | 4.5  (-1.03 to 9.03) | 1  (-2.5  to 6.5) | 2.5  (-1.29 to 6.29) |
| P700 S300 | 100  (92.45 to 103.55) | 95  (30.19 to 125.81) | 100  (92.43 to 103.55) | 1389  (-390.62 to 7151.82) | 1243 (172.19 to 3993.41) | 1306 (266.55 to 4168.65) | 15  (3.67 to 34.33) | 6  (0.92 to 16.28) | 11  (2.92 to 15.08) |
| P700 S200 | 88  (62.09 to 103.57) | 87.5  (59.3 to 103.36) | 90  (45.07 to 108.27) | 815  (363.3 to 1315.36) | 604  (420.53 to 749.47) | 551.5  (-99.65 to 1662.32) | 3  (0.48 to 8.52) | 1.5  (-0.2 to 3.87) | 0  (constant) |
| P500 S400 | 27.5  (-10.14 to 62.64) | 22.5  (-8.31 to 53.31) | 10  (-20.1 to 55.1) | 250  (-317.35 to 985.35) | 268.5  (-249.11 to 835.11) | 251  (-316.48 to 984.48) | 0  (-1.09 to 2.09) | 0  (-0.55 to 1.05) | 0  (-2.18 to 4.18) |
| P500 S300 | 60  (48.43 to 74.43) | 45  (33.44 to 57.41) | 40  (21.09 to 57.48) | 1390 (110.59 to 5540.55) | 1199 (478.45 to 1641.83) | 821  (71.18 to 2351.40) | 9  (3.25 to 17.89) | 4  (0.33 to 5.39) | 0  (-1.24 to 2.95) |
| P500 S200 | 60  (41.43 to 75.14) | 63  (34.17 to 68.4) | 60  (25.64 to 72.93) | 890  (-187.08 to 3091.36) | 645  (53.6 to 1850.68) | 720  (122.22 to 2097.78) | 8  (2.75 to 12.96) | 4  (1.44 to 7.99) | 4  (-0.42 to 11.56) |
