# Supplementary material for: Determining the porous structure for optimal soft-tissue ingrowth: An in vivo histological study
Source: PLoS One. 2018 Oct 29;13(10):e0206228. doi: 10.1371/journal.pone.0206228 (PMC6205611; doi:10.1371/journal.pone.0206228)
Supplement: S3 Table — (DOCX) [file pone.0206228.s003.docx]

S3 Table. Zone 2 - Percentage Fill p values

Kruskall Wallis p value=0.000

|  | P1000  S400 | P1000  S200 | P700  S400 | P700  S300 | P700  S200 | P500  S400 | P500  S300 | P500  S200 |
| --- | --- | --- | --- | --- | --- | --- | --- | --- |
| P1000  S400 |  | 0.731 | 0.010 |  |  | 0.010 |  |  |
| P1000  S200 |  |  |  |  | 0.138 |  |  | 0.001 |
| P700  S400 |  |  |  | 0.190 | 0.010 | 0.343 |  |  |
| P700  S300 |  |  |  |  | 0.792 |  | 0.106 |  |
| P700  S200 |  |  |  |  |  |  |  | 0.051 |
| P500  S400 |  |  |  |  |  |  | 0.073 | 0.073 |
| P500  S300 |  |  |  |  |  |  |  | 0.383 |
| P500  S200 |  |  |  |  |  |  |  |  |
