# Supplementary material for: Determining the porous structure for optimal soft-tissue ingrowth: An in vivo histological study
Source: PLoS One. 2018 Oct 29;13(10):e0206228. doi: 10.1371/journal.pone.0206228 (PMC6205611; doi:10.1371/journal.pone.0206228)
Supplement: S7 Table — (DOCX) [file pone.0206228.s007.docx]

S7 Table. Zone 3 - Cell nuclei count/mm^2^ p values

Kruskall Wallis test p=0.000

|  | P1000  S400 | P1000  S200 | P700  S400 | P700  S300 | P700  S200 | P500  S400 | P500  S300 | P500  S200 |
| --- | --- | --- | --- | --- | --- | --- | --- | --- |
| P1000  S400 |  | 0.073 | 0.067 |  |  | 0.038 |  |  |
| P1000  S200 |  |  |  |  | 0.002 |  |  | 0.001 |
| P700  S400 |  |  |  | 0.016 | 0.914 | 0.686 |  |  |
| P700  S300 |  |  |  |  | 0.030 |  | 0.149 |  |
| P700  S200 |  |  |  |  |  |  |  | 0.101 |
| P500  S400 |  |  |  |  |  |  | 0.109 | 0.109 |
| P500  S300 |  |  |  |  |  |  |  | 0.902 |
| P500  S200 |  |  |  |  |  |  |  |  |
