# Supplementary material for: Determining the porous structure for optimal soft-tissue ingrowth: An in vivo histological study
Source: PLoS One. 2018 Oct 29;13(10):e0206228. doi: 10.1371/journal.pone.0206228 (PMC6205611; doi:10.1371/journal.pone.0206228)
Supplement: S9 Table — (DOCX) [file pone.0206228.s009.docx]

S9 Table. Zone 2 - Blood vessel count/mm^2^ p values

Kruskall Wallis test p=0.005

|  | P1000  S400 | P1000  S200 | P700  S400 | P700  S300 | P700  S200 | P500  S400 | P500  S300 | P500  S200 |
| --- | --- | --- | --- | --- | --- | --- | --- | --- |
| P1000  S400 |  | 0.534 | 0.114 |  |  | 0.010 |  |  |
| P1000  S200 |  |  |  |  | 0.008 |  |  | 0.165 |
| P700  S400 |  |  |  | 0.063 | 0.914 | 0.486 |  |  |
| P700  S300 |  |  |  |  | 0.009 |  | 0.048 |  |
| P700  S200 |  |  |  |  |  |  |  | 0.138 |
| P500  S400 |  |  |  |  |  |  | 0.230 | 0.024 |
| P500  S300 |  |  |  |  |  |  |  | 0.456 |
| P500  S200 |  |  |  |  |  |  |  |  |
