# Supplementary material for: Determining the porous structure for optimal soft-tissue ingrowth: An in vivo histological study
Source: PLoS One. 2018 Oct 29;13(10):e0206228. doi: 10.1371/journal.pone.0206228 (PMC6205611; doi:10.1371/journal.pone.0206228)
Supplement: S11 Table — (DOCX) [file pone.0206228.s011.docx]

**S11 Table. Correlations**

| Spearman’s rho | | | Percentage soft tissue fill | | | Cell nuclei/mm^2^ | | | Blood vessel/mm^2^ | | |
| --- | --- | --- | --- | --- | --- | --- | --- | --- | --- | --- | --- |
|  |  |  | Zone 1 | Zone 2 | Zone 3 | Zone 1 | Zone 2 | Zone 3 | Zone 1 | Zone 2 | Zone 3 |
| Percentage soft tissue fill | Zone 1 | Correlation Coefficient |  | 0.877 | 0.941 | 0.620 |  |  | 0.323 |  |  |
|  |  | Sig (2-tailed) |  | 0.000 | 0.000 | 0.000 |  |  | 0.031 |  |  |
|  | Zone 2 | Correlation Coefficient |  |  | 0.868 |  | 0.610 |  |  | 0.493 |  |
|  |  | Sig (2-tailed) |  |  | 0.000 |  | 0.000 |  |  | 0.001 |  |
|  | Zone 3 | Correlation Coefficient |  |  |  |  |  | 0.621 |  |  | 0.432 |
|  |  | Sig (2-tailed) |  |  |  |  |  | 0.000 |  |  | 0.003 |
| Cell nuclei  /mm^2^ | Zone 1 | Correlation Coefficient |  |  |  |  | 0.799 | 0.880 | 0.316 |  |  |
|  |  | Sig (2-tailed) |  |  |  |  | 0.000 | 0.000 | 0.035 |  |  |
|  | Zone 2 | Correlation Coefficient |  |  |  |  |  | 0.765 |  | 0.635 |  |
|  |  | Sig (2-tailed) |  |  |  |  |  | 0.000 |  | 0.000 |  |
|  | Zone 3 | Correlation Coefficient |  |  |  |  |  |  |  |  | 0.503 |
|  |  | Sig (2-tailed) |  |  |  |  |  |  |  |  | 0.000 |
| Blood vessel  /mm^2^ | Zone 1 | Correlation Coefficient |  |  |  |  |  |  |  | 0.760 | 0.333 |
|  |  | Sig (2-tailed) |  |  |  |  |  |  |  | 0.000 | 0.025 |
|  | Zone 2 | Correlation Coefficient |  |  |  |  |  |  |  |  | 0.629 |
|  |  | Sig (2-tailed) |  |  |  |  |  |  |  |  | 0.000 |
|  | Zone 3 | Correlation Coefficient |  |  |  |  |  |  |  |  |  |
|  |  | Sig (2-tailed) |  |  |  |  |  |  |  |  |  |
