# Supplementary material for: Determining the porous structure for optimal soft-tissue ingrowth: An in vivo histological study
Source: PLoS One. 2018 Oct 29;13(10):e0206228. doi: 10.1371/journal.pone.0206228 (PMC6205611; doi:10.1371/journal.pone.0206228)
Supplement: S12 Table — (DOCX) [file pone.0206228.s012.docx]

**S12 Table. Intraclass Correlation Coefficient Data for Semi-quantitative Percentage Soft Tissue Fill Score**

|  | **Median value (and 95% Confidence Interval)** | |
| --- | --- | --- |
|  | Observer 1 | Observer 2 |
| P1000 S400 | 100.00 (90.86 to 98.58) | 100.00(96.06 to 100.83) |
| P1000 S200 | 90.00 (84.03 to 94.30) | 100.00 (94.23 to 99.66) |
| P700 S400 | 75.00 (59.19 to 82.47) | 30.00 (24.14 to 45.19) |
| P700 S300 | 100.00 (92.06 to 99.94) | 100.00 (94.59 to 100.08) |
| P700 S200 | 85.00 (76.18 to 92.71) | 87.50 (68.95 to 91.60) |
| P500 S400 | 35.00 (16.91 to 48.09) | 35.00 (15.50 to 47.00) |
| P500 S300 | 50.00 (38.34 to 55.95) | 50.00 (36.55 to 56.12) |
| P500 S200 | 60.00 (45.45 to 62.17) | 60.00 (43.69 to 62.22) |

| **Reliability Statistics** | |
| --- | --- |
| Cronbach's Alpha | N of Items |
| .932 | 2 |

| **Intraclass Correlation Coefficient** | | | | | | | |
| --- | --- | --- | --- | --- | --- | --- | --- |
|  | Intraclass Correlation^b^ | 95% Confidence Interval | | F Test with True Value 0 | | | |
|  |  | Lower Bound | Upper Bound | Value | df1 | df2 | Sig |
| Single Measures | .870^a^ | .822 | .906 | 14.673 | 134 | 134 | .000 |
| Average Measures | .931^c^ | .902 | .951 | 14.673 | 134 | 134 | .000 |
| Two-way mixed effects model where people effects are random and measures effects are fixed. | | | | | | | |
| a. The estimator is the same, whether the interaction effect is present or not. | | | | | | | |
| b. Type A intraclass correlation coefficients using an absolute agreement definition. | | | | | | | |
| c. This estimate is computed assuming the interaction effect is absent, because it is not estimable otherwise. | | | | | | | |
